# Oral Squamous Papilloma of the Lower Labial Mucosa: A Case Report

**DOI:** 10.7759/cureus.106734

**Published:** 2026-04-09

**Authors:** Aneena Shakeer, Priya K Nair, Renju Jose, Mahija Janardhanan, Aravind M S

**Affiliations:** 1 Oral Medicine and Radiology, Amrita School of Dentistry, Amrita Vishwa Vidyapeetham University, Kochi, IND; 2 Oral Pathology and Microbiology, Amrita School of Dentistry, Amrita Vishwa Vidyapeetham University, Kochi, IND

**Keywords:** excisional biopsy, human papillomavirus, oral squamous papilloma, tobacco smoking, verrucopapillary lesions

## Abstract

Oral squamous papilloma (OSP) is a benign epithelial proliferation commonly encountered in the oral cavity and often presents as an exophytic growth with a papillary surface architecture. Although typically non-aggressive, its clinical resemblance to other verrucopapillary lesions necessitates careful diagnostic evaluation.

This report describes a case of OSP in a 58-year-old male who presented to a tertiary care center in Kerala, India, with a history of chronic tobacco smoking and an incidental exophytic lesion on the lower labial mucosa. The lesion was surgically excised using a diode laser, and histopathological examination confirmed the diagnosis.

Early recognition and histopathological confirmation are essential for appropriate management and to rule out potentially malignant lesions, especially in patients with risk-modifying habits such as tobacco use.

## Introduction

Oral squamous papilloma (OSP) is a benign proliferative lesion of stratified squamous epithelium characterized by papillary or verruciform exophytic growth [1]. It represents one of the most common benign epithelial tumors of the oral cavity and accounts for approximately 2-4% of all biopsied oral lesions [2]. Clinically, it presents as a solitary, slow-growing, painless mass with a pedunculated or sessile base and a characteristic cauliflower-like surface morphology [3].

The etiopathogenesis of OSP is strongly associated with human papillomavirus (HPV), particularly low-risk genotypes HPV-6 and HPV-11 [4]. HPV is a double-stranded deoxyribonucleic acid (DNA) virus with epithelial tropism that infects basal keratinocytes through micro-abrasions in the mucosa [5]. Viral replication is closely linked to epithelial differentiation, resulting in hyperplastic epithelial proliferation and the formation of papillary projections [6]. Unlike high-risk HPV types (16 and 18), which integrate into host DNA and disrupt tumor suppressor pathways such as p53 and pRb, low-risk types typically remain episomal and are associated with benign lesions [7].

Recent literature has increasingly emphasized the clinical relevance of HPV in oral lesions rather than detailed molecular mechanisms, particularly in benign conditions such as OSP. The reported prevalence of HPV in oral papillomas varies widely depending on detection methods and population characteristics, with studies from Asian populations demonstrating comparable but heterogeneous trends. In addition to viral factors, local environmental influences such as tobacco exposure play an important modifying role, contributing to epithelial alterations that may facilitate viral persistence and lesion development. In regions such as India, where tobacco use is prevalent, these factors are particularly relevant in interpreting oral mucosal lesions [5,8,9].

Histopathologically, OSP demonstrates finger-like projections of hyperparakeratinized stratified squamous epithelium supported by fibrovascular connective tissue cores [8]. Koilocytosis, characterized by perinuclear halos and nuclear hyperchromasia, may be observed, reflecting HPV-induced cytopathic changes [9]. Mitotic activity is generally confined to the basal layer, and cellular atypia is absent in typical cases [10].

Epidemiologically, OSP can occur across a broad age range but is most commonly reported in adults between the third and sixth decades of life [2,11]. There is no consistent gender predilection, though some studies report slight female predominance [1]. In pediatric populations, OSP constitutes approximately 7-8% of oral tumors [12]. While lesions in adults are typically solitary, multiple lesions may be observed more frequently in children and immunocompromised individuals [13,14]. This variation highlights differences in host immune response and viral behavior across age groups, contributing to distinct clinical presentations.

Common intraoral sites include the tongue, soft palate, uvula, lips, and labial mucosa [15-17]. Despite its benign nature, the clinical significance of OSP lies in its resemblance to other verrucopapillary lesions, necessitating careful differential diagnosis. Lesions such as verruca vulgaris, condyloma acuminatum, focal epithelial hyperplasia, verruciform xanthoma, papillary hyperplasia, and early verrucous carcinoma may present with overlapping clinical features, making histopathological confirmation essential [18,19].

Risk factors contributing to OSP development include HPV exposure, tobacco use, alcohol consumption, chronic irritation, and immunosuppression [15]. Tobacco smoking, in particular, has been associated with increased epithelial susceptibility to viral persistence and mucosal alterations such as melanosis and keratinization [16]. Although malignant transformation of OSP is exceedingly rare, persistent HPV infection and synergistic carcinogenic factors warrant careful evaluation [17].

Management primarily involves complete surgical excision with narrow margins [20]. Alternative treatment modalities include laser ablation, electrocautery, and cryotherapy [21]. Recurrence is uncommon and typically associated with incomplete removal or persistent viral infection [22]. Prognosis is excellent in immunocompetent individuals [3].

The present report describes a case of OSP in a chronic smoker, emphasizing clinical features, diagnostic approach, histopathological correlation, and management considerations.

## Case presentation

A 58-year-old male patient reported to a tertiary care center in Kochi, Kerala, India, with the chief complaint of multiple decayed teeth in the lower posterior region for one year and missing teeth in both upper and lower arches for three to four years. The patient’s primary concern was a loosened tooth and prosthetic rehabilitation. Medical and family history were non-contributory. The patient reported a history of smoking 20 cigarettes per day for approximately 13 years and occasional alcohol consumption. No history of systemic illness or immunocompromised state was elicited. Extraoral examination revealed no significant changes.

**Figure 1:**
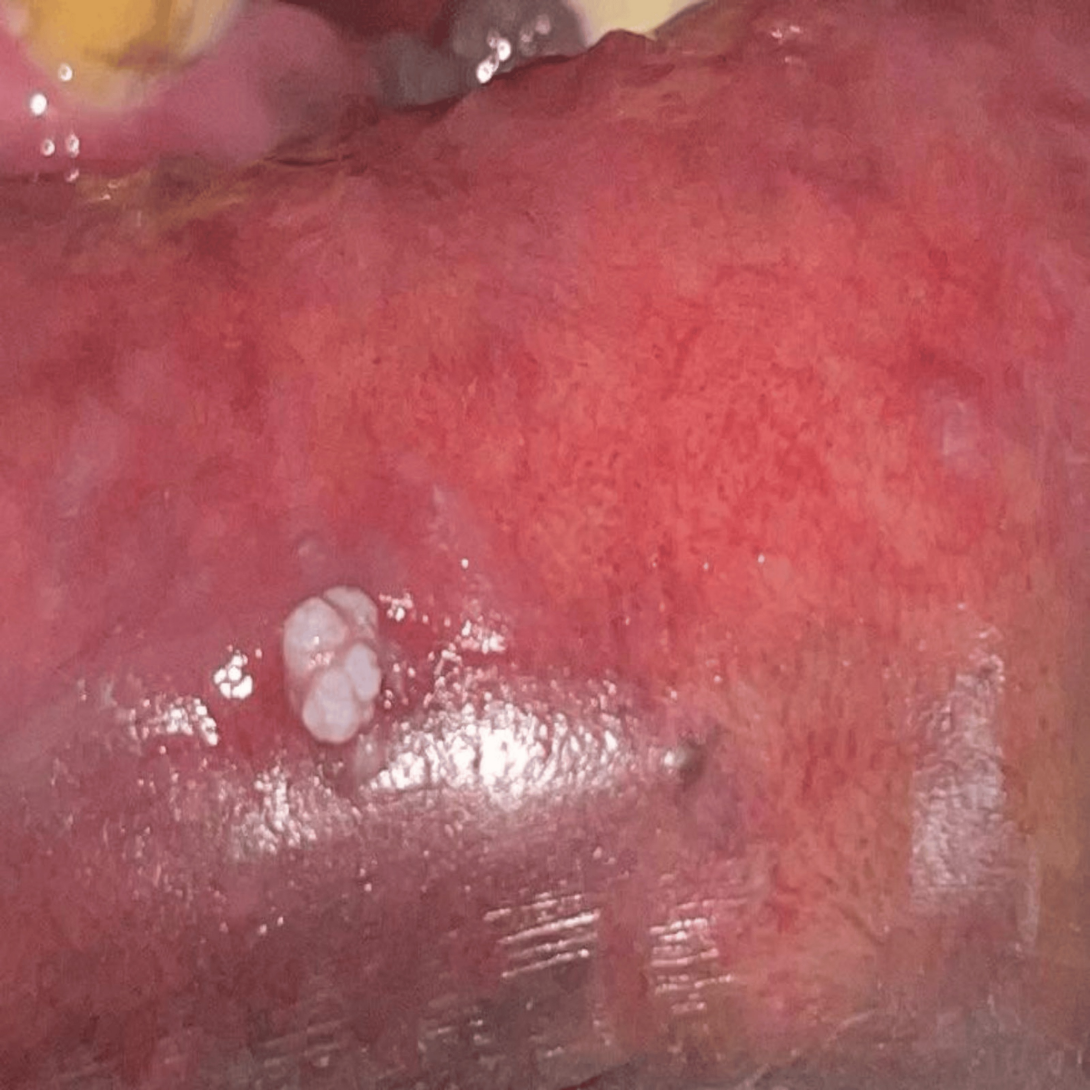
Clinical appearance of the lesion on the lower labial mucosa A solitary, well-defined, pedunculated exophytic lesion with a cauliflower-like surface morphology is seen on the lower labial mucosa in relation to teeth 31 and 41. The lesion appears non-ulcerated and non-erythematous, with surrounding mucosa showing features of smoker’s melanosis. Picture credits: Dr. Aneena Shakeer

Intraoral examination revealed diffuse greyish-black pigmentation, suggestive of smoker’s melanosis on the lower labial mucosa. Incidentally, a solitary exophytic growth was observed on the lower labial mucosa in relation to teeth 31 and 41. The lesion measured approximately 0.5 × 0.6 cm. It was pedunculated, non-tender, non-scrapable, and exhibited a cauliflower-like surface morphology. No bleeding, ulceration, or discharge was present. The surrounding mucosa appeared otherwise normal except for pigmentation. The clinical presentation is outlined in Figure 1.

Based on clinical presentation, a provisional diagnosis of squamous papilloma was made. Differential diagnoses included verruca vulgaris, condyloma acuminatum, focal epithelial hyperplasia, and early verrucous carcinoma.

Routine hematological investigations were performed before the biopsy. Table 1 summarizes the patient’s preoperative hematological parameters. All values were within normal physiological limits.

**Table 1:** Preoperative hematological parameters of the patient, including measured values and corresponding normal reference ranges mg/dL: milligrams per deciliter

Parameter	Patient Value	Reference Range
Random Blood Sugar	97 mg/dL	70-140 mg/dL
Bleeding Time	2 minutes 30 seconds	2-7 minutes
Clotting Time	4 minutes 30 seconds	3-8 minutes

**Figure 2:**
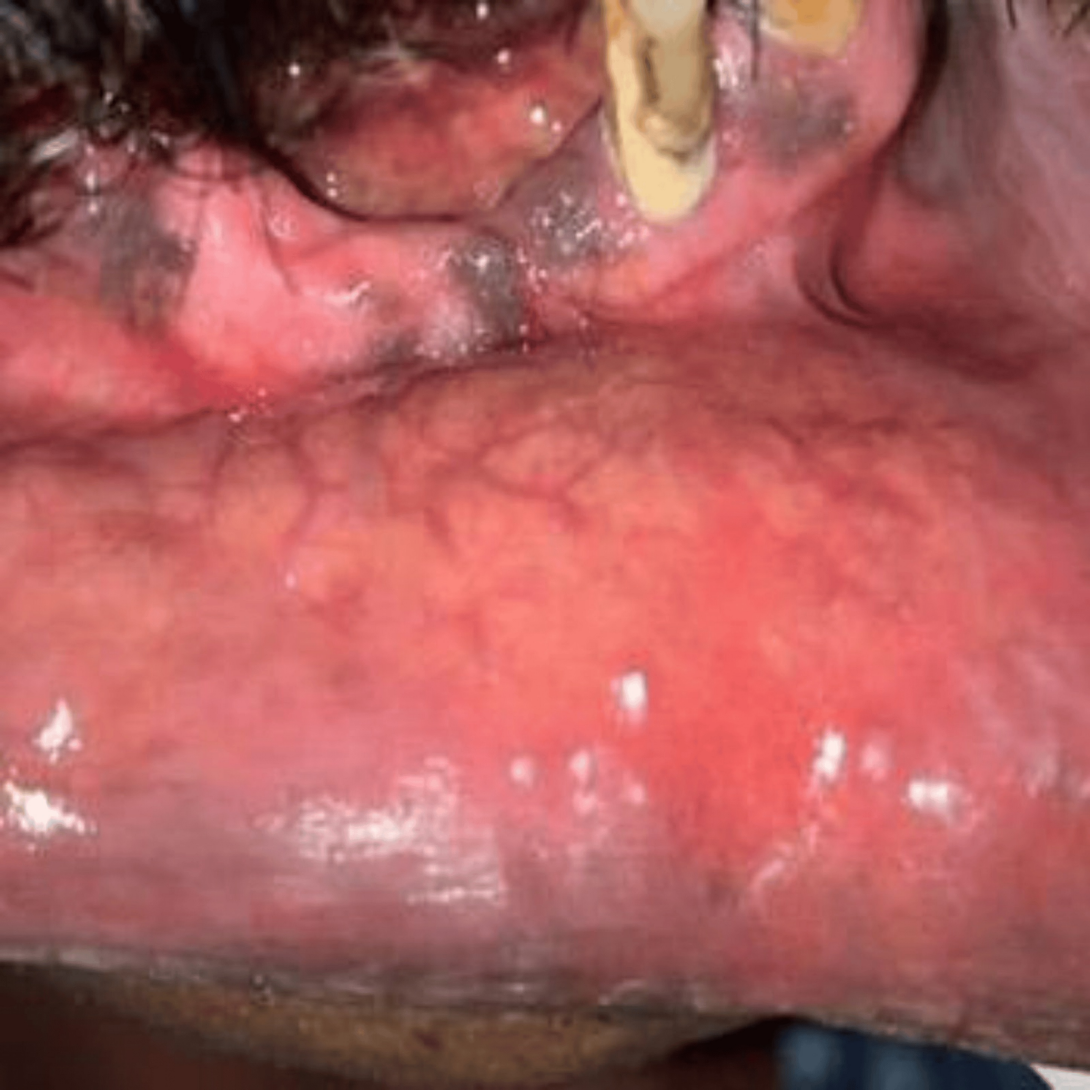
Post-excisional surgical site Postoperative view of the lower labial mucosa following diode laser excision, showing satisfactory wound healing with no evidence of bleeding, infection, or residual lesion. Picture credits: Dr. Aneena Shakeer

An excisional biopsy was planned. Under local anesthesia, the lesion was completely excised using a diode laser, which offers advantages such as reduced intraoperative bleeding, better visibility, and minimal postoperative discomfort. The surgical site was irrigated with povidone-iodine and saline. Suturing was performed to approximate wound margins. This is shown in Figure 2.

**Figure 3:**
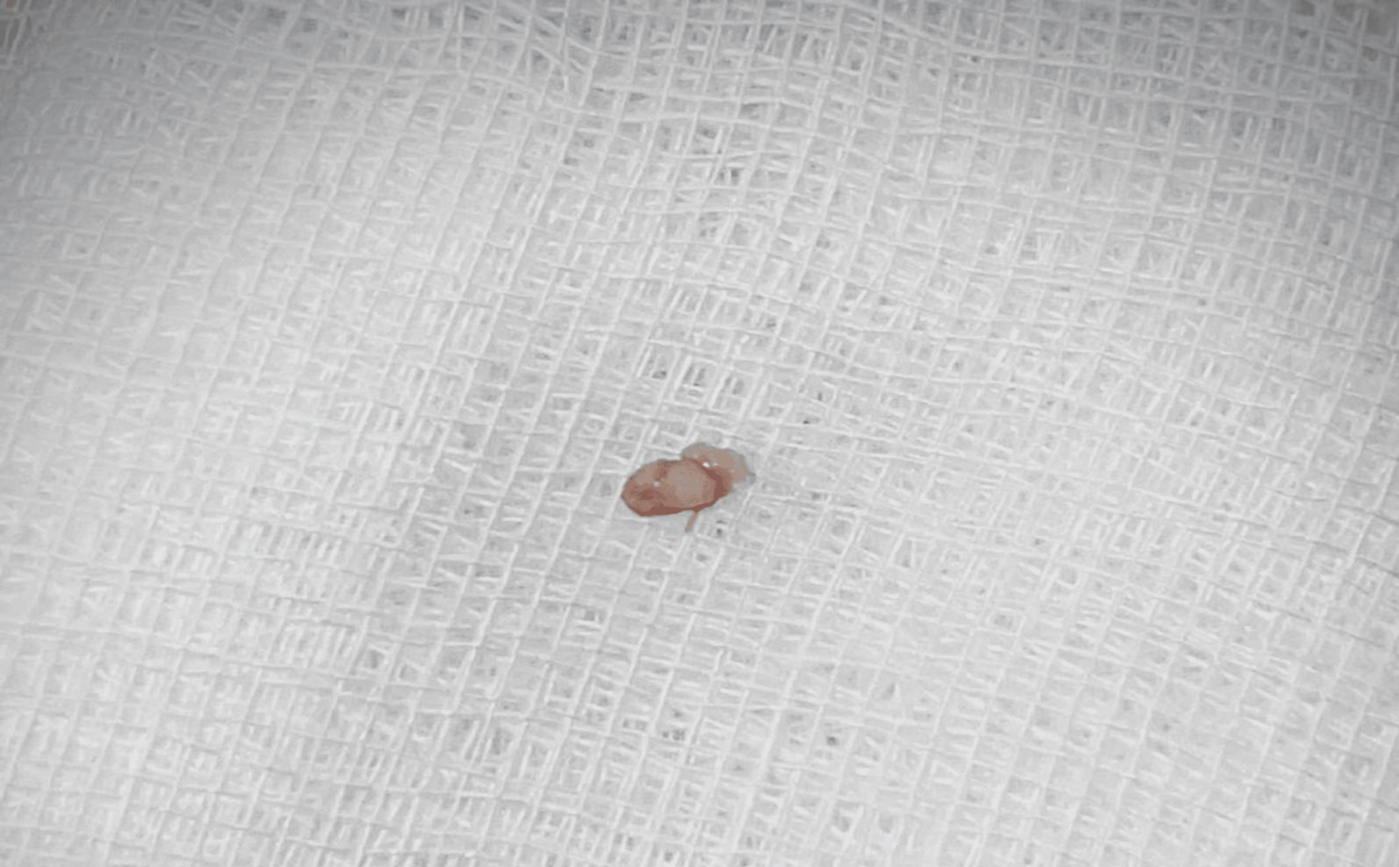
Excised specimen Gross specimen of the excised lesion placed on sterile gauze, showing a small, soft tissue mass with papillary surface features consistent with a clinical diagnosis of oral squamous papilloma. Picture credits: Dr. Aneena Shakeer

The excised specimen was submitted for histopathological evaluation. This is shown in Figure 3.

**Figure 4:**
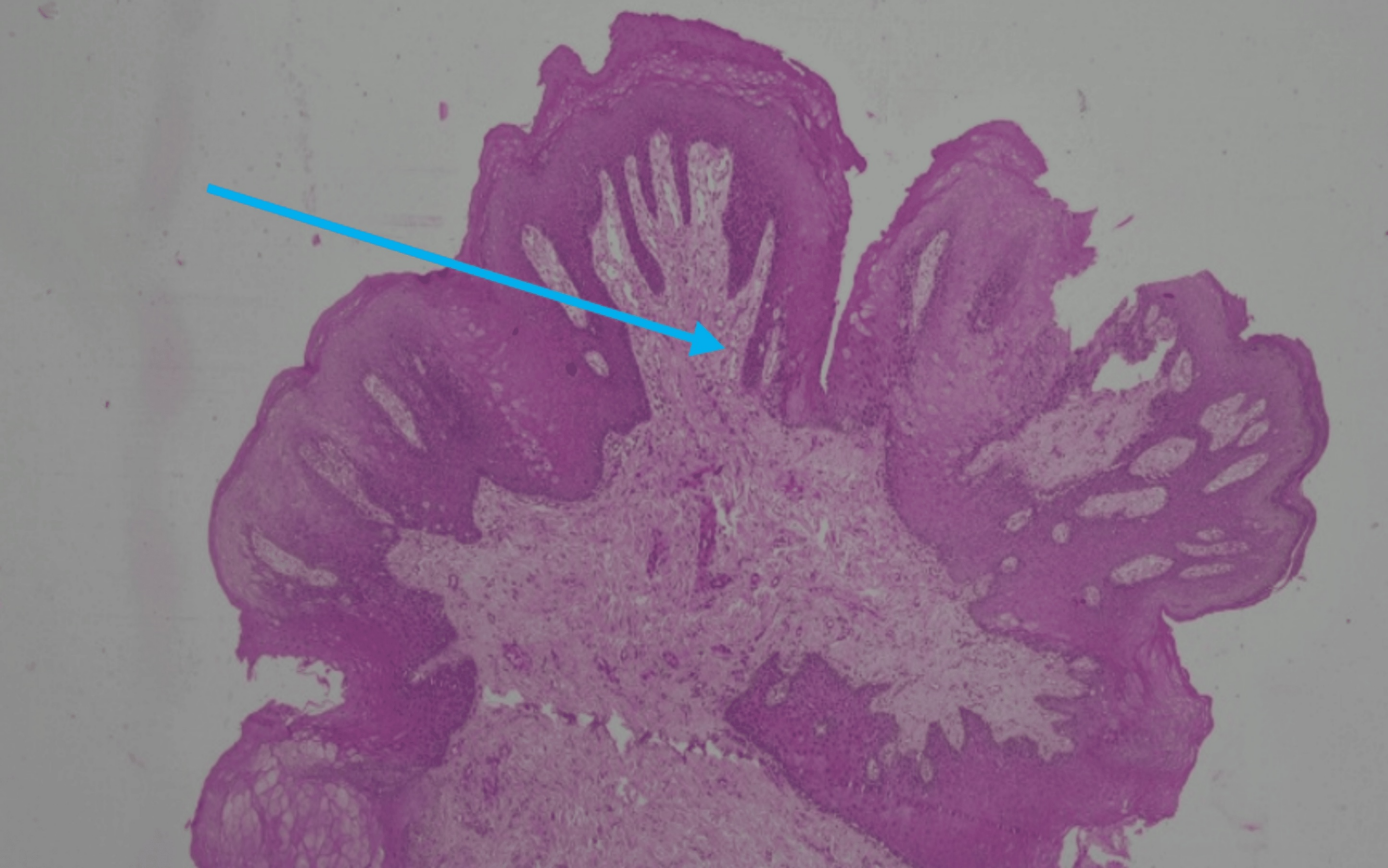
Low-power view (scanning magnification) H&E-stained section at 4x magnification illustrating a pedunculated exophytic growth. The lesion exhibits multiple finger-like papilliferous surface projections composed of hyperplastic stratified squamous epithelium. Each projection is supported by a thin, central core of vascularized fibrovascular connective tissue (blue arrow) extending from the underlying lamina propria. Picture credits: Dr. Mahija Janardhanan

**Figure 5:**
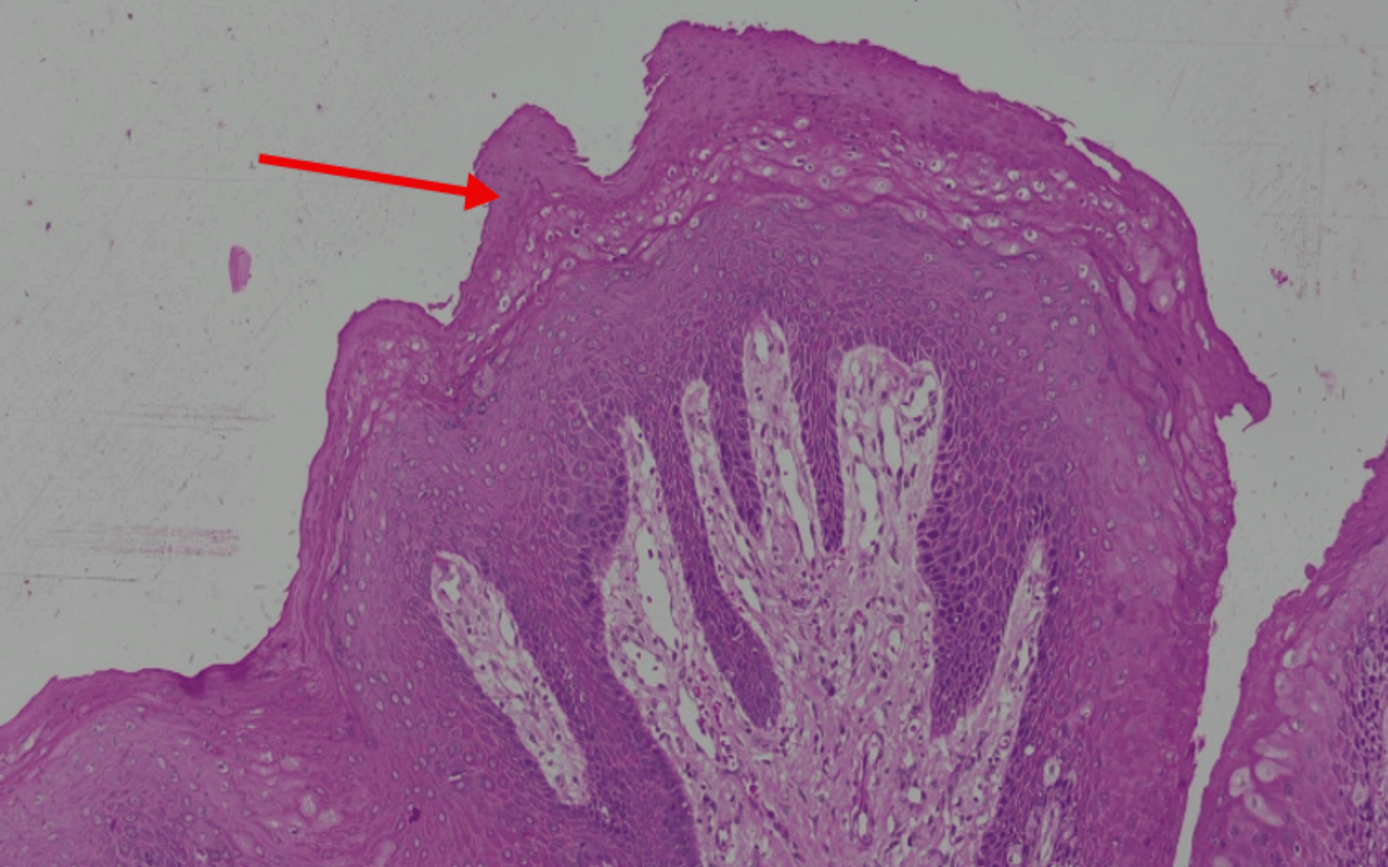
Intermediate-power view H&E-stained section at 20x magnification showing the structural detail of the papillary stalks. The epithelium displays prominent acanthosis and a well-defined basal cell layer. The fibrovascular connective tissue is continuous with the stalk of the lesion, while the papilliferous surface (red arrow) shows evidence of orthokeratosis or parakeratosis, typical of oral squamous papilloma. Picture credits: Dr. Mahija Janardhanan

**Figure 6:**
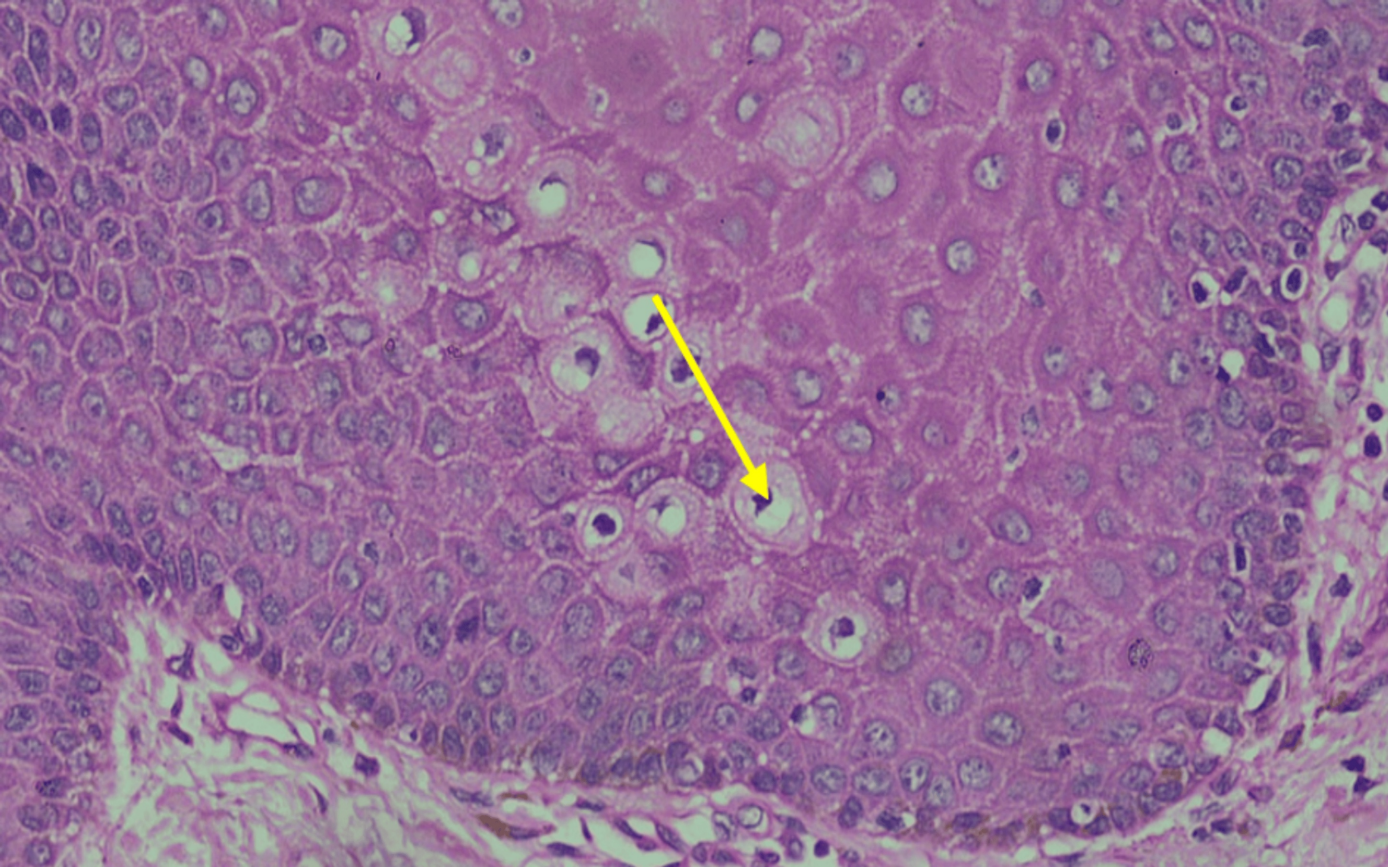
High-power view (cellular detail) H&E-stained section at 40x magnification, focusing on the spinous and granular layers of the epithelium. Numerous koilocytes (yellow arrow) are visible, characterized by pyknotic, hyperchromatic nuclei surrounded by a clear perinuclear cytoplasmic halo. Koilocytic changes observed within the epithelium are suggestive of HPV-associated cytopathic effect; however, definitive confirmation requires molecular diagnostic techniques such as polymerase chain reaction (PCR), in situ hybridization (ISH), or immunohistochemistry. Picture credits: Dr. Mahija Janardhanan

Upon microscopic examination, an exophytic lesion characterized by hyperplastic superficial stratified squamous epithelium thrown into numerous papilliferous surface projections was seen. The epithelium shows parakeratosis. A prominent granular layer was noticed. Focal koilocytic change was noticed within the epithelium. No evidence of dysplasia or malignant transformation was observed, confirming the diagnosis of OSP. The histopathologic representation is shown in Figures 4-6.

**Figure 7:**
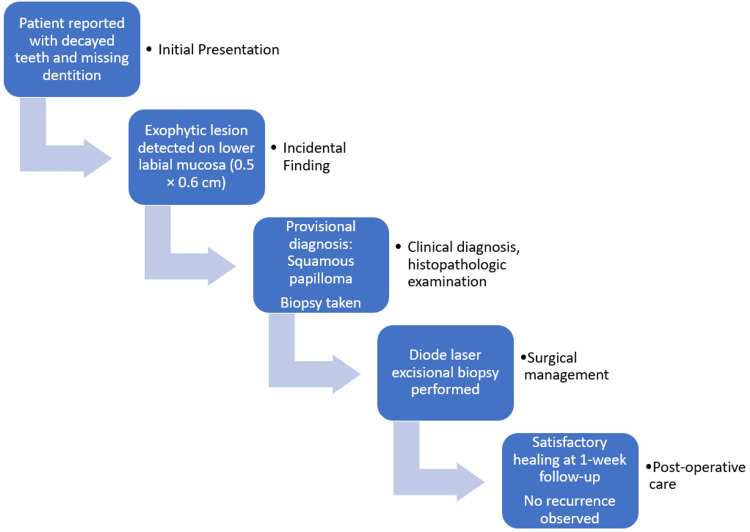
Clinical timeline illustrating the patient’s course from initial presentation to postoperative follow-up This figure illustrates the sequence of clinical events, including initial presentation with dental complaints, incidental detection of the lesion, excisional biopsy using a diode laser, and satisfactory postoperative healing. The timeline was created using Microsoft PowerPoint (Microsoft Corporation, Redmond, WA, USA).

Postoperative medications included analgesics and an antiseptic mouth rinse for seven days. The patient was advised to undergo smoking cessation counselling. Follow-up at one week showed satisfactory healing, and complete mucosal recovery was observed subsequently. No recurrence was noted. The clinical timeline is detailed in Figure 7.

## Discussion

OSP is a benign epithelial neoplasm characterized by papillary proliferation of stratified squamous epithelium, most commonly associated with low-risk HPV genotypes [1,4]. It constitutes a significant proportion of oral epithelial proliferations and remains an important diagnostic consideration in verrucopapillary lesions of the oral cavity [2]. Although biologically benign, its clinical resemblance to potentially malignant disorders needs careful evaluation.

The etiopathogenesis of OSP is closely linked to HPV infection, particularly genotypes 6 and 11 [4]. These low-risk viral types infect basal keratinocytes through micro-abrasions in the mucosal epithelium and replicate in synchrony with epithelial differentiation [6]. The viral genome persists episomally rather than integrating into host DNA, which explains the non-aggressive biological behavior of the lesion [7]. The E6 and E7 oncoproteins produced by high-risk HPV strains (16 and 18) are known to inactivate tumor suppressor proteins p53 and retinoblastoma (pRb), facilitating oncogenesis [17]. However, such oncogenic integration is rarely observed in solitary OSP, reinforcing its benign nature.

In the present case, the lesion was identified while evaluating decayed teeth. This aligns with the typical clinical presentation of OSP, which is often asymptomatic and slow-growing [3]. Abbey et al. analyzed 464 cases and reported that most lesions were less than 1 cm in diameter and predominantly solitary [2]. The size of the lesion in our patient (0.5 × 0.6 cm) falls within this commonly reported range. The lower labial mucosa, though less frequently involved than the tongue and soft palate, remains a recognized site [13].

The patient’s history of chronic tobacco smoking is clinically significant. Tobacco exposure induces epithelial hyperplasia, keratinization, and pigmentation changes such as smoker’s melanosis [15,16]. Chronic exposure to tobacco carcinogens may compromise local immune surveillance and epithelial integrity, potentially facilitating viral persistence [9]. While smoking itself is not a direct etiologic factor for OSP, it acts as a modifying cofactor in epithelial pathology and increases the overall risk of malignant transformation in the oral cavity [15]. Therefore, histopathological confirmation is mandatory in smokers presenting with verrucous lesions.

The role of tobacco smoking in OSP warrants further clinical consideration. While tobacco is not a direct etiological agent in the development of OSP, it functions as a significant modifying cofactor by altering the local mucosal microenvironment. Chronic exposure to tobacco smoke leads to epithelial hyperkeratinization, increased mucosal permeability, and microvascular changes, which may facilitate viral entry and persistence. Additionally, tobacco-related immunomodulation impairs local immune surveillance by reducing Langerhans cell activity and altering cytokine profiles, thereby diminishing the host’s ability to clear HPV infection effectively [9,16]. This compromised immune response may prolong viral persistence and contribute to lesion development or delayed resolution. Therefore, in patients with a history of tobacco use, OSP should be interpreted within a broader context of epithelial vulnerability and increased oncogenic risk, necessitating careful histopathological evaluation and long-term monitoring.

Differential diagnosis remains critical due to overlapping clinical features among verrucopapillary lesions. Verruca vulgaris, commonly caused by HPV-2 and HPV-4, typically exhibits more pronounced hyperkeratosis and elongated rete ridges converging toward the center [18]. Condyloma acuminatum, usually associated with sexual transmission, often presents as multiple lesions with broader and blunter papillary projections [19]. Verrucous carcinoma and early exophytic squamous cell carcinoma must be excluded due to their malignant potential; these lesions exhibit invasive growth patterns, dysplasia, and pushing borders histologically [18]. Table 2 summarizes key distinguishing features to aid in the clinical and histopathological differentiation of common verrucopapillary lesions of the oral cavity.

**Table 2:** Comparative clinical and histopathological features of oral squamous papilloma, verruca vulgaris, and condyloma acuminatum Table credits: Dr. Aneena Shakeer

Feature	Oral Squamous Papilloma	Verruca Vulgaris	Condyloma Acuminatum
Etiology	HPV-6, HPV-11	HPV-2, HPV-4	HPV-6, HPV-11 (sexual transmission)
Number of lesions	Usually solitary	Usually solitary	Often multiple
Common sites	Tongue, soft palate, lips	Skin, vermilion border, oral mucosa	Labial mucosa, lingual frenum, soft palate
Clinical appearance	Pedunculated, cauliflower-like	Papillary, rough, hyperkeratotic	Sessile, broader, blunted papillary projections
Size	Usually <1 cm	Variable	Often larger than a papilloma
Transmission	Non-sexual	Autoinoculation	Sexual contact
Histopathology	Finger-like projections with fibrovascular cores; koilocytosis	Hyperkeratosis, elongated rete ridges converging centrally	Broad-based projections with acanthosis and koilocytosis
Malignant potential	Extremely rare	None	Low but higher than papilloma

Microscopic examination of hematoxylin and eosin-stained sections revealed a well-circumscribed, exophytic lesion composed of hyperplastic stratified squamous epithelium arranged in multiple finger-like papilliferous projections. Each projection was supported by a central fibrovascular connective tissue core continuous with the underlying lamina propria [9,22]. The surface epithelium exhibited predominantly parakeratinization, with focal areas of orthokeratinization. A prominent granular cell layer and acanthosis were evident. The rete ridges appeared elongated and mildly pointed, without dysplastic architectural disturbance. Focal koilocytic changes were observed within the superficial epithelial layers, characterized by perinuclear clearing and nuclear hyperchromasia. These features are suggestive of HPV-associated cytopathic effect, although not diagnostic in the absence of molecular confirmation [10].

The basement membrane remained intact, with no evidence of epithelial invasion into the underlying connective tissue. Mitotic figures were sparse and confined to the basal layer, with no atypical mitoses identified. The underlying connective tissue stroma showed a mild chronic inflammatory infiltrate composed predominantly of lymphocytes. Importantly, no features of epithelial dysplasia or malignant transformation were observed. Based on these findings, a definitive diagnosis of OSP was established [23].

Laser excision was chosen in this case due to its well-documented advantages. Laser surgery provides superior hemostasis, reduced postoperative edema, decreased bacterial contamination, and minimal scarring [21]. Studies have demonstrated favorable healing outcomes and low recurrence rates following laser excision of benign oral lesions [21]. Conventional scalpel excision remains the gold standard and is equally effective when complete removal with adequate margins is achieved [20]. Recurrence rates for solitary OSP are low, generally attributed to incomplete excision or persistent viral infection [22].

Malignant transformation of solitary OSP is exceedingly rare [17]. However, persistent HPV infection, immunosuppression, and synergistic carcinogenic exposures such as tobacco and alcohol may theoretically increase oncogenic risk [24,25]. Gillison et al. highlighted the growing role of HPV in oropharyngeal carcinomas, particularly HPV-16 [24,26]. Although the pathogenic pathways differ between low-risk and high-risk HPV types, clinicians must remain vigilant when evaluating papillomatous lesions in high-risk individuals.

The present case reinforces the importance of comprehensive oral examination, even when patients present for unrelated dental complaints. Incidental findings can represent clinically significant pathology requiring intervention. Although OSP is a common benign lesion, this case is notable for its incidental detection in a chronic smoker with adjacent smoker’s melanosis, highlighting the importance of thorough oral examination even in patients presenting with unrelated complaints. The case also emphasizes the role of tobacco-induced epithelial alterations as a potential modifying factor in lesion development. Furthermore, successful management using diode laser excision demonstrates a minimally invasive approach with favorable clinical outcomes. Thus, the significance of this report lies in its contextual presentation in a high-risk individual, its incidental discovery, and its effective management, reinforcing the need for clinical vigilance in routine dental practice [25,26].

From a public health perspective, HPV-related oral lesions underscore the broader implications of viral oncogenesis. Prophylactic HPV vaccination has demonstrated efficacy in reducing infection with high-risk genotypes and may potentially reduce the incidence of HPV-associated oral lesions in the future [25,26]. Although vaccination primarily targets cervical and anogenital HPV infection, its expanding role in head and neck oncology is increasingly recognized.

Smoking cessation counseling was advised for this patient to mitigate the overall oral cancer risk. Tobacco cessation not only reduces epithelial dysplastic changes but also enhances mucosal immunity and wound healing. Regular follow-up remains essential to monitor for recurrence or development of additional lesions.

In summary, OSP is a benign HPV-associated epithelial proliferation with characteristic clinical and histological features. Despite its non-aggressive behavior, accurate differentiation from potentially malignant lesions is critical. Complete excision, histopathological confirmation, patient education, and risk factor modification collectively ensure optimal prognosis.

Limitations

This case report has certain limitations that must be acknowledged. First, the absence of definitive HPV confirmation through molecular diagnostic methods such as polymerase chain reaction (PCR), in situ hybridization (ISH), or immunohistochemistry (IHC) limits the ability to establish a direct etiological association between the lesion and HPV. Although histopathological features such as koilocytosis were observed, these findings are suggestive but not diagnostic of HPV infection. Second, while the histopathological evaluation was comprehensive, advanced ancillary investigations, such as immunohistochemical markers, were not performed. Inclusion of such markers could have provided additional insight into epithelial proliferative activity and helped further differentiate benign from potentially dysplastic or malignant lesions. Third, it represents a single-patient observation, which limits the generalizability of the findings. Fourth, although short-term postoperative healing was satisfactory, long-term follow-up data beyond the initial mucosal recovery period were not available, restricting assessment of recurrence or late complications. Additionally, advanced diagnostic modalities such as HPV typing or immunohistochemical analysis were not performed, which could have provided further insight into the viral characteristics and biological behavior of the lesion. Future studies with larger sample sizes and extended follow-up are necessary to better understand the clinical implications and recurrence patterns of OSP.

## Conclusions

OSP is a benign HPV-associated epithelial proliferation that may clinically mimic other verrucopapillary lesions. Accurate diagnosis requires thorough clinical examination and histopathological confirmation. Complete surgical excision provides definitive treatment with an excellent prognosis. In patients with tobacco exposure, careful evaluation and habit counseling are essential to reduce potential oncogenic risk. Early detection remains the cornerstone of optimal management.
